# Oral Administration of Germinated, Pigmented, Giant Embryo Rice (*Oryza sativa* L. cv. Keunnunjami) Extract Improves the Lipid and Glucose Metabolisms in High-Fat Diet-Fed Mice

**DOI:** 10.1155/2021/8829778

**Published:** 2021-01-15

**Authors:** Soo Im Chung, Mi Young Kang

**Affiliations:** ^1^International Agricultural Training Center, Kyungpook National University, Daegu 41566, Republic of Korea; ^2^Department of Food Science and Nutrition, Kyungpook National University, Daegu 41566, Republic of Korea

## Abstract

Obesity is a significant risk factor for chronic diseases. The effect of ethanol extract from germinated Keunnunjami, blackish-purple rice with a giant embryo, compare to ordinary brown rice, on the body weight and lipid and glucose metabolism in high-fat diet-fed mice was analyzed. Mice were fed with a high-fat diet-fed for 3 weeks and then orally administered with either distilled water (HF) or extract (0.25%, *w*/*w*) from brown, germinated brown, Keunnunjami, and germinated Keunnunjami rice for 4 weeks. Control mice were fed with a normal diet and orally administered with distilled water. The HF group showed markedly higher body weight and triglyceride, cholesterol, fatty acid, glucose, and insulin levels than the control group. However, the oral administration of rice extracts ameliorated this high-fat diet-induced obesity, hyperlipidemia, and hypoglycemia through the modulation of adipokine production, lipogenic and glucose-regulating enzyme activities, and mRNA expression of genes associated with lipid and glucose metabolism. The germinated Keunnunjami extract exhibited greater hypolipidemic, hypoglycemic, and body weight-lowering effects than the other rice extracts. The results demonstrated that germination could further enhance the physiological properties of rice and that germinated Keunnunjami extract has a strong therapeutic potential against high-fat diet-induced obesity, hyperlipidemia, and hyperglycemia.

## 1. Introduction

Obesity, dyslipidemia, and diabetes have become worldwide health problems due to their increasing prevalence in developed and developing countries. According to the World Health Organization, in 2016, more than 1.9 billion adults worldwide were overweight, and more than 650 million were obese. In 2008, 39% of adults worldwide have elevated total cholesterol (TC) [1, 2]. Diabetes was the ninth leading cause of death in 2019 [3], and its global incidence among adults rose from 108 million in 1980 to 422 million in 2014 [4]. Studies have revealed that chronic consumption of a high-fat diet accompanied by a sedentary lifestyle could cause obesity, dyslipidemia, and diabetes, resulting in an increased risk of developing cardiovascular disease [5–8]. With the rapidly increasing incidence of these metabolic disorders, therapeutic intervention has become more urgent. Natural drugs and food products that can regulate lipid and glucose metabolism and have a body weight-lowering effect may help prevent and treat of high-fat diet-induced obesity, dyslipidemia, and diabetes.

Rice grains with colored brans, known as pigmented varieties, have been reported to reduce triglyceride, cholesterol, and glucose levels in rats and mice [9, 10]. Similarly, rice varieties with an enlarged or giant embryo exhibited hypoglycemic and hypocholesterolemic effects in high-fat diet-fed mice and high cholesterol-fed rats [11–13]. Pigmented rice and giant embryo rice are rich in bioactive compounds such as polyphenols, *γ*-aminobutyric acid (GABA), and *γ*-oryzanol, which have various pharmacological properties [14–16]. Dietary feeding of black rice with giant embryo has been shown to ameliorate obesity and significantly decrease glucose, insulin, and triacylglycerol levels in *ob/ob* mice [17]. Germination causes complex biochemical changes that results in increased nutrients and bioactive components in germinated rice [18]. Such changes include the activation of dormant enzymes associated with the synthesis of bioactive compounds and the breaking down of the cell wall, leading to the release of bound and free materials [19, 20]. Previous studies have revealed that germinated rice contains higher phytochemical levels than nongerminated rice and has strong hypolipidemic and antidiabetic activities [19, 21].

Keunnunjami is a new specialty rice cultivar with blackish-purple pigmented bran and a giant embryo developed through conventional breeding in Korea. Keunnunjami contains higher amounts of bioactive compounds and has greater antiobesity, hypolipidemic, and antioxidant activities than ordinary brown rice [22–24]. Moreover, diet supplementation of germinated Keunnunjami rice powder has been recently reported to improve lipid metabolism in ovariectomized rats [25]. Because Keunnunjami rice is a newly developed functional rice, information on its biological activities is still limited. Further studies on the effect of germination on the physiological properties of Keunnunjami are needed to understand its therapeutic potential against metabolic diseases better. Hence, this study was carried out to determine the effects of ethanol extracts from germinated and nongerminated Keunnunjami rice, in compare to those from ordinary nonpigmented normal embryo brown rice, on lipid and glucose metabolism in mice under high-fat diet condition.

## 2. Materials and Methods

### 2.1. Rice Germination and Ethanol Extract Preparation

The Department of Agricultural Science of Korea National Open University provided whole grains of Ilpum rice, a nonpigmented normal embryo cultivar, and Keunnunjami rice, a blackish-purple pigmented cultivar with giant embryo. The two rice samples were cultivated from May to September 2016 in Dangjin, Chungcheongnam-do, South Korea, and germinated based on the method of Wu et al. [26] with slight modifications. Briefly, 50 g of rice grains was washed with distilled water and placed evenly in a tray overlaid with cotton pads and cheesecloth. The tray was added with distilled water (100 ml), covered with a plastic wrap with holes, and incubated at 30°C in an oven for 72 h. The rice grains were checked every 12 h to ensure that no foul odor and fungal growth occurred. The germinated grains were then dried at 50°C for 2 hr and ground using a grinding machine (HMF-3250S; Hanil Electronics, Seoul, South Korea). The rice powder was passed through a 50 to 100 mesh sieve and extracted with 70% ethanol (1 : 3 solid/solvent ratio) at 40°C with constant stirring for 2 h. The mixture was filtered, and the residue was extracted again with 70% ethanol (1 : 1 solid to solvent ratio) at 40°C with constant stirring. The process was repeated, and the extracts were combined and concentrated using a rotary evaporator (Eyela N-1000, Tokyo, Japan) to remove the ethanol, obtaining an extract yield of 5% (*w*/*w*). For the nongerminated rice samples, 50 g of rice grains was washed, dried, ground, and extracted using the same method described above for the germinated grains. The germinated and nongerminated rice extracts were freeze-dried, and their bioactive compounds such as octacosanol, quercetin, ferulic acid, GABA, and *γ*-oryzanol were analyzed using previously described methods [27–30]. The results are presented in Table 1. All chemicals and reagents used in this study were of analytical grade and purchased from Merck KGaA (Darmstadt, Hessen, Germany) and Sigma-Aldrich, Inc. (St. Louis, MO, USA).

### 2.2. Animals and Diets

Sixty male C57BL/6N mice aged 4 weeks old and each weighing approximately 20 g were purchased from Central Laboratory Animal, Inc. (Seoul, Korea). Each animal was housed in a hanging stainless steel cage placed in a room maintained at 25°C ± 2°C with 50% relative humidity and 12 h light-12 h dark cycle. Mice were initially fed with a commercial pelletized diet and distilled water ad libitum for 1 week and then randomly divided into two groups. The first group (*n* = 10; NC group) was fed with a normal control AIN-76 diet [31]. The second group (*n* = 50) was fed with a high-fat diet (45% calories from fat, lard) purchased from Feed Lab (Gurisi, KyungGi-do, Korea) to induce obesity. After 3 weeks, obese mice were randomly divided into five dietary groups (*n* = 10) and administered via oral gavage with either distilled water (HF group) or ethanol extracts from normal brown rice (HF-B group), germinated brown rice (HF-GB group), Keunnunjami rice (HF-K group), or germinated Keunnunjami rice (HF-GK group) at a dose of 0.25% (*w*/*w*) [32]. The NC group was orally administered with distilled water. After 4 weeks, all animals were anesthetized with carbon dioxide by inhalation after a 12 h fast. The blood samples were drawn from the inferior vena cava into a heparin-coated tube and centrifuged at 1000 × *g* for 15 min at 4°C to obtain the plasma. The liver, heart, kidney, and white adipose tissues (epididymal, perirenal, inguinal) were removed, rinsed with physiological saline, weighed, and stored at −70°C until analysis. This study protocol was approved by the Ethics Committee of Kyungpook National University for animal studies (approval no. KNU. 2016-0025).

### 2.3. Determination of Lipid Profile

The plasma total triglyceride (TG), TC, and high-density lipoprotein cholesterol (HDL-C) levels were determined using commercial kits (Asan Pharmaceutical, Gyeonggi-do, Korea). The plasma free fatty acid content was measured using a commercial assay kit (Enzychrom; Bioassay Systems, Hayward, CA, USA). The apolipoprotein (Apo) A-I and B contents were analyzed using an enzyme-linked immunosorbent assay (ELISA) kit (MyBioSource, San Diego, CA, USA). The plasma aspartate aminotransferase (AST) and alanine aminotransaminase (ALT) levels were determined using commercial kits (Sigma-Aldrich).

### 2.4. Determination of Glucose, Insulin, and Adipokine Levels

The blood glucose level was measured using a glucose assay (AM201-K) kit (Asan Pharmaceutical). The plasma insulin content was determined using an ELISA kit (TMB Mouse Insulin ELISA kit; Shibayagi Co., Gunma, Japan). The plasma adiponectin, resistin, and tumor necrosis factor-*α* (TNF-*α*) levels were analyzed using adipokine ELISA kits (Shibayagi Co.). The homeostasis index of insulin resistance (HOMA-IR) was calculated according to the mathematical formula [33]: HOMA − IR = [fasting insulin (*μ*lU/ml) × fasting glucose (mmol/L)]/22.5.

### 2.5. Analysis of Lipid- and Glucose-Regulating Enzymes and *β*-Oxidation Activities

The activities of hepatic and adipocyte lipid-regulating enzymes, such as fatty acid synthase (FAS), glucose-6-phosphate dehydrogenase (G6PD), and carnitine palmitoyltransferase (CPT), and *β*-oxidation, were analyzed based on the method of Park et al. [34]. The activities of hepatic and kidney glucose-regulating enzymes, such as glucokinase (GK), phosphoenolpyruvate carboxykinase (PEPCK), and glucose-6-phosphatase (G6pase), were determined according to previously described methods [35].

### 2.6. Analysis of mRNA Gene Expression

Total RNA was isolated from liver tissue (0.1 g) using TRIzol reagent (Invitrogen, Carlsbad, CA, USA) according to the manufacturer's instructions. The isolated RNA was converted to cDNA using the High Capacity RNA-to-cDNA kit (Applied Biosystems, Foster City, CA, USA). The mRNA expression of FAS (FASN), peroxisome proliferator-activated receptor-*γ* (PPAR-*γ*), glucose-dependent insulinotropic polypeptide (GIP), and small heterodimer partner interacting leucine zipper protein (SMILE) was quantified by the Step One Real-Time PCR System (Applied Biosystems) using TaqMan Gene Expression Assays (Applied Biosystems Ltd., Tokyo, Japan). Glyceraldehyde 3-phosphate dehydrogenase, a housekeeping gene, was used as the control for mRNA expression analysis.

### 2.7. Statistical Analysis

Data are presented as the mean ± standard error of the mean (SEM). Data were evaluated by one-way analysis of variance (SPSS 19.0; SPSS, Inc., Chicago, IL, USA). The differences between the means were assessed using Tukey's range test. Statistical significance was considered at *p* < 0.05.

## 3. Results

### 3.1. Body Weight Gain

The initial body weights were similar in all mice groups (Table 2). At the end of the experiment, the HF group showed substantially higher weight gain and total body fat than NC mice. In contrast, all rice extract-administered groups, particularly HF-GK mice, exhibited significantly lower weight gain and body fat than the HF group. The feed intake and feed efficiency ratio were highest in HF mice. A significant increase in the liver, heart, and kidney weights was also observed in the HF group.

### 3.2. Plasma Lipid Profile

The TG, TC, and FFA levels markedly increased, whereas the HDL-C content decreased in HF mice relative to the NC group (Table 3). Moreover, the HF group showed a significantly lower HDL-C/TC ratio (HTR) and higher atherogenic index (AI) than control mice. However, all rice extract-administered groups exhibited substantially lower TG, TC, and FFA contents and AI and higher HDL-C level and HTR than HF mice. Apo B, AST, and ALT concentrations increased, whereas the Apo A-I content and Apo A-I/Apo B ratio decreased with a high-fat diet. In contrast, oral administration of rice extracts significantly decreased Apo B, AST, and ALT levels and increased the Apo A-I level and Apo A-I/Apo B ratio in high-fat diet-fed mice. In general, ethanol extracts from germinated Keunnunjami and brown rice showed greater lipid-lowering effects than those from their nongerminated counterparts. HF-GK mice exhibited the highest hypolipidemic activity among the rice extract-fed groups.

### 3.3. Glucose, Insulin, and Adipokine Levels

The initial blood glucose levels were the same across all groups (Table 4). The high-fat diet resulted in a significant increase in the final glucose level, plasma insulin content, and HOMA-IR. The adipokine resistin and TNF-*α* levels also increased, whereas the adiponectin content decreased in HF mice relative to the NC group. Administration of rice extracts counteracted high-fat diet-induced elevation of glucose, insulin, resistin, and TNF-*α* levels and reduction of adiponectin concentration. Among the rice extract-administered groups, HF-GK mice showed the lowest glucose, insulin, resistin, TNF-*α* levels and highest adiponectin content and HOMA-IR followed by the HF-K and HF-GB groups.

### 3.4. Lipid-Regulating Enzyme Activities

HF mice exhibited the highest activities of hepatic and adipocyte lipid-regulating enzymes FAS and G6PD and lowest CPT enzyme and *β*-oxidation activities (Table 5). HF-GK mice showed significantly lower FAS and G6PD activities and higher CPT and *β*-oxidation activities than the other rice extract-fed groups.

### 3.5. Glucose-Regulating Enzyme Activities

HF mice exhibited a marked increase in the activities of hepatic and nephritic enzymes PEPCK and G6pase and a decrease in GK enzyme activity relative to the NC group (Table 6). In contrast, rice extract-administered groups, especially HF-GK mice, showed significantly lower PEPCK and G6pase activities and higher GK activity than HF mice.

### 3.6. Relative mRNA Levels of FASN, PPAR-*γ*, GIP, and SMILE

**Figure 1:**
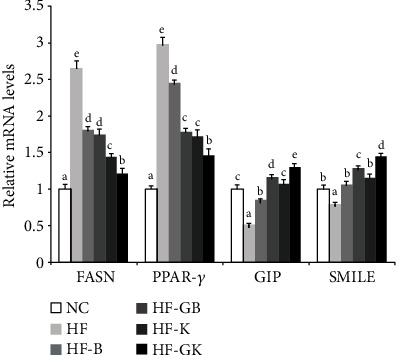
Relative mRNA expression of hepatic FASN, PPAR-*γ*, GIP, and SMILE genes in high-fat diet-fed mice orally administered with germinated and nongerminated Keunnunjami and brown rice extracts. Means not sharing a common superscript are significantly different at *p* < 0.05 (*n* = 10).

The mRNA expression levels of FASN and PPAR-*γ* considerably increased, whereas those of GIP and SMILE significantly decreased with high-fat feeding (Figure 1). However, oral administration of rice extracts counteracted high-fat diet-induced FASN and PPAR-*γ* upregulation and GIP and SMILE downregulation. HF-GK mice showed the lowest FASN and PPAR-*γ* mRNA expression levels and highest GIP and SMILE expression levels among the rice extract-administered animal groups.

## 4. Discussion

This study analyzed the effect of oral administration of ethanol extract from Keunnunjami rice (blackish-purple pigmented cultivar with giant embryo) germinated for 72 h, compared to that of Ilpum (nonpigmented cultivar with normal embryo), on the body weight and lipid and glucose metabolism in high-fat diet-fed mice. The results showed that high-fat feeding significantly increased body weight gain, body fat, and TC, TG, FFA, glucose, and insulin levels in mice. In contrast, oral administration of rice extracts attenuated high-fat diet-induced body weight gain, hyperlipidemia, and hyperglycemia. Moreover, HF mice showed markedly lower HDL-C and Apo A-I levels, HTR, and Apo A-I/Apo B ratio and substantially higher AI and Apo B, AST, and ALT contents than the NC and rice extract-administered groups. Apo A-I is the primary protein component in HDL particles, which have antiatherogenic property, and is associated with a reduced risk of coronary heart disease. Apo B is the major protein constituent in low-density lipoprotein particles, which have atherogenic property, and is correlated with an increased risk of coronary heart disease [36, 37]. HTR, AI, and Apo A-I/Apo B ratio are considered risk indicators of cardiovascular disease. Decreased AI and increased HTR and Apo A-I/Apo B ratio in HF-B, HF-GB, HF-K, and HF-GK groups relative to that of HF mice suggested that rice extracts could significantly lower the risk of cardiovascular disease. AST and ALT enzymes are specific markers of liver damage, and their decreased levels in rice extract-administered groups relative to the HF group indicate a reduction in high-fat diet-induced hepatic oxidative stress in these animals. Previous investigations also revealed that dietary feeding of Keunnunjami rice powder inhibited body weight gain and hyperlipidemia in high-fat diet-fed mice, and diet supplementation of germinated Keunnunjami rice powder improved lipid metabolism in ovariectomized rats [24, 25]. Extracts from germinated black rice have also been shown to reduce plasma glucose, insulin, cholesterol, and triglyceride levels in diabetic rats [38]. Similarly, dietary intake of reddish-brown rice powder improved glucose metabolism in ovariectomized rats, and germinated rice showed a greater hypoglycemic effect than nongerminated rice [23]. In this study, germinated Keunnunjami and brown rice extracts showed greater hypolipidemic, hypoglycemic, and body weight-lowering effects than their nongerminated counterparts, indicating that germination for 72 h could enhance the physiological properties of the rice samples. Among the rice extract-administered groups, HF-GK mice exhibited the lowest body weight gain, body fat, TG, TC, FFA, glucose, insulin, HOMA-IR, AI, and Apo A-I/Apo B ratio and highest HDL-C level and HTR, suggesting that the germinated Keunnunjami rice extract has superior physiological functions than the other rice extract samples.

The body weight-lowering effect and hypolipidemic and hypoglycemic activities of germinated and nongerminated rice extracts were probably associated with decreased resistin and TNF-*α* levels; reduced activities of FAS, G6PD, PEPCK, and G6pase enzymes; increased adiponectin level and CPT, *β*-oxidation, and GK activities; downregulation of FASN and PPAR-*γ* mRNA expression; and upregulation of GIP and SMILE expression in obese mice. Adipokine adiponectin, resistin, and TNF-*α* are protein hormones that regulate lipid and glucose metabolism [39]. Adiponectin has antiatherosclerotic, hepatoprotective, and insulin-sensitizing properties and is inversely correlated with obesity, insulin resistance, and diabetes [40, 41]. Resistin and TNF-*α* are directly related to the progression of obesity and diabetes [42, 43]. Lipogenic enzymes FAS, G6PD, and CPT are involved in the biosynthesis of cholesterol and fatty acid, and increased FAS and G6PD activities have been associated with lipid dysregulation, insulin resistance, and elevated triglyceride levels [44, 45]. An increase in CPT activity, however, promotes fatty acid oxidation, resulting in reduced triglyceride level diabetes [46]. Glucose-regulating enzymes GK, PEPCK, and G6pase are involved in the regulation of glucose metabolism. GK enzyme is associated with glucose homeostasis, and its enhanced activity has been linked to decreased blood glucose levels [47]. PEPCK and G6pase enzymes are involved in gluconeogenesis, and their increased activities are associated with increased glucose production [48, 49]. PPAR-*γ* is a nuclear receptor that regulates adipogenesis, fatty acid storage, and glucose metabolism, and its inhibition has been shown to ameliorate high-fat diet-induced obesity and diabetes [50, 51]. GIP is a gut hormone that modulates glucose-dependent insulin secretion and improves glucose tolerance in obese diabetic rats [52, 53]. SMILE is an insulin-inducible corepressor that regulates hepatic gluconeogenesis, and its increased expression has been shown to improve hepatic lipid metabolism and ameliorate hyperglycemia and glucose intolerance in high-fat diet-fed mice [54, 55]. Hence, reduction in body weight gain and body fat and decrease in TG, TC, free fatty acid, glucose, and insulin levels in high-fat diet-fed mice orally administered with sample extracts, especially those from germinated rice, may have been through a mechanism involving the regulation of adipokine production and lipogenic and glucose-regulating enzyme activities and modulation of the FASN, PPAR-*γ*, GIP, and SMILE expression.

Germination causes complex biochemical changes that result in increased nutrients and bioactive components in germinated rice [18]. Such changes include the activation of dormant enzymes associated with the synthesis of bioactive compounds and the breaking down of the cell wall, leading to the release of bound and free materials [19, 20]. Rather than analyze the effect of one effective substance on metabolism, this study is aimed at identifying whether lipid and glucose metabolism is improved through complex interactions of increased bioactive compounds that are generally readily edible. In this study, germination for 72 h significantly increased the amount of bioactive compounds, such as octacosanol, quercetin, ferulic acid, GABA, and *γ*-oryzanol, in Keunnunjami rice. The brown rice sample had increased ferulic acid, GABA, and *γ*-oryzanol levels after germination. These phytochemicals are known to possess several physiological functions. For instance, GABA, the most generated compound during rice germination, has anticancer, antiobesity, antidiabetic, hepatoprotective, antihyperlipidemic, and antihypertensive properties [16, 18, 56]. GABA is considered one of the active compounds responsible for the cholesterol-lowering effect of germinated giant embryo rice [13]. *γ*-Oryzanol, also one of the main bioactive components in germinated rice, and ferulic acid, the major phenolic compound in rice, have strong antioxidant and anticarcinogenic properties [15, 26, 57] and have been shown to improve lipid and glucose metabolism in high-fat diet-fed mice [58, 59]. Similarly, octacosanol has been found to ameliorate hyperlipidemia in diabetic mice and improved lipid metabolism in high-fat diet-fed mice [60, 61]. Quercetin has also been found to possess antiobesity, antidiabetic, and antioxidative properties [62, 63]. The increased amount of bioactive compounds during germination may have been responsible for the greater body weight-lowering effect and hypolipidemic and hypoglycemic activities of germinated rice extracts, particularly Keunnunjami rice, compared to nongerminated rice extracts.

## 5. Conclusion

Pigmented rice is one of the most widely consumed food ingredients and is abundant in active physiological substances. Also, germinated pigmented rice with fortified functional substances is closely related to improving health and includes phenolics, flavanones, tocols, policosanols, and anthocyanins, among which quercetin, ferulic acid, GABA, and *γ*-oryzanols are the major functional component responsible for biological activities.

Oral administration of blackish-purple rice Keunnunjami and ordinary brown rice ethanol extracts significantly reduced body weight gain and improved lipid and glucose profiles in obese mice through a mechanism involving the modulation of adipokine production, activities of lipid- and glucose-regulating enzymes, and mRNA expression of genes associated with lipid and glucose metabolism. Germination for 72 h further enhanced the hypolipidemic, hypoglycemic, and body weight-lowering effects of Keunnunjami and brown rice, which may have been due to the increase in the levels of bioactive compounds. Ethanol extract from germinated Keunnunjami rice showed greater physiological activities than that from germinated ordinary brown rice. These findings illustrated that germination can improve the functional properties of Keunnunjami rice and that ethanol extracts from germinated Keunnunjami rice may have a strong therapeutic potential against obesity, hyperlipidemia, and hyperglycemia caused by a high-fat diet.

## Figures and Tables

**Table 1:** Bioactive compounds in germinated and nongerminated Keunnunjami and brown rice extracts.

Bioactive compound (mg/100 g extract)	Brown rice		Keunnunjami	
	Nongerminated	Germinated	Nongerminated	Germinated
Octacosanol	2.47 ± 0.07^a^	2.55 ± 0.05^a^	2.79 ± 0.09^b^	4.34 ± 0.00^c^
Quercetin	0.01 ± 0.00^a^	0.01 ± 0.01^a^	1.64 ± 0.02^b^	2.14 ± 0.03^c^
Ferulic acid	1.27 ± 0.02^a^	3.01 ± 0.01^b^	5.87 ± 0.09^c^	12.19 ± 0.08^d^
GABA	24.47 ± 0.01^a^	711.22 ± 4.74^c^	87.14 ± 3.24^b^	989.87 ± 1.87^d^
*γ*-Oryzanol	22.14 ± 0.07^a^	33.19 ± 2.91^b^	34.58 ± 2.49^b^	62.87 ± 4.05^c^

Mean ± SEM (*n* = 3). Means in the same row with different letters are significantly different at *p* < 0.05 by Tukey's range test.

**Table 2:** Body weight gain and organ weights of high-fat diet-fed mice orally administered with germinated and nongerminated Keunnunjami and brown rice extracts.

Parameter	Animal group					
	NC	HF	HF-B	HF-GB	HF-K	HF-GK
Initial body weight (g)	20.40 ± 0.14^a^	20.35 ± 0.10^a^	20.37 ± 0.13^a^	20.29 ± 0.13^a^	20.28 ± 0.05^a^	20.36 ± 0.04^a^
Final body weight (g)	27.36 ± 0.09^a^	39.22 ± 0.30^d^	35.68 ± 0.36^c^	32.16 ± 0.34^b^	32.74 ± 0.23^b^	30.75 ± 0.38^b^
Body weight gain (g/day)	0.21 ± 0.01^a^	0.57 ± 0.01^f^	0.45 ± 0.01^e^	0.37 ± 0.01^d^	0.34 ± 0.01^c^	0.31 ± 0.01^b^
Feed intake (g/day)	2.81 ± 0.05^a^	3.38 ± 0.02^c^	2.99 ± 0.04^b^	2.84 ± 0.04^a^	2.83 ± 0.02^a^	2.82 ± 0.08^a^
Feed efficiency ratio	0.08 ± 0.00^a^	0.16 ± 0.00^e^	0.14 ± 0.00^d^	0.12 ± 0.00^c^	0.12 ± 0.00^c^	0.10 ± 0.00^b^
Liver (g/100 g)	3.81 ± 0.02^a^	6.89 ± 0.02^e^	6.75 ± 0.03^d^	6.04 ± 0.04^c^	6.01 ± 0.03^c^	5.01 ± 0.11^b^
Heart (g)	0.46 ± 0.02^a^	0.51 ± 0.01^b^	0.48 ± 0.01^a^	0.46 ± 0.01^a^	0.47 ± 0.00^a^	0.45 ± 0.02^a^
Kidney (g)	1.15 ± 0.02^a^	1.23 ± 0.02^b^	1.19 ± 0.02^ab^	1.21 ± 0.02^ab^	1.20 ± 0.02^ab^	1.16 ± 0.02^a^
Total body fat (g)	7.01 ± 0.25^a^	14.27 ± 0.27^e^	11.53 ± 0.19^d^	10.58 ± 0.15^c^	10.31 ± 0.23^c^	9.20 ± 0.13^b^

Mean ± SEM (*n* = 10). Means in the same row with different letters are significantly different at *p* < 0.05 by Tukey's range test. NC: AIN-76 diet; HF: high-fat diet; HF-B: HF + normal brown rice extract; HF-GB: HF + germinated normal brown rice extract; HF-K: HF + Keunnunjami rice extract; HF-GK: HF + germinated Keunnunjami rice extract.

**Table 3:** Plasma lipid profile in high-fat diet-fed mice orally administered with germinated and nongerminated Keunnunjami and brown rice extracts.

Parameter	Animal group					
	NC	HF	HF-B	HF-GB	HF-K	HF-GK
TG (mg/dl)	120.54 ± 1.25^a^	156.72 ± 0.98^e^	142.99 ± 1.67^d^	131.30 ± 1.20^b^	137.82 ± 1.09^c^	128.24 ± 1.76^b^
TC (mg/dl)	145.18 ± 3.42^a^	265.37 ± 5.56^e^	243.93 ± 2.25^d^	197.60 ± 3.01^c^	200.68 ± 2.34^c^	177.53 ± 0.94^b^
HDL-C (mg/dl)	53.67 ± 1.43^e^	32.88 ± 0.79^a^	38.15 ± 0.48^b^	47.98 ± 0.86^d^	43.15 ± 0.71^c^	48.31 ± 0.84^d^
HTR (%)	36.98 ± 0.89^e^	12.39 ± 0.06^a^	15.69 ± 0.71^b^	24.28 ± 0.14^c^	16.62 ± 0.33^b^	27.22 ± 0.67^d^
AI	1.71 ± 0.07^a^	7.07 ± 0.04^f^	5.23 ± 0.28^e^	3.19 ± 0.03^c^	3.65 ± 0.12^d^	2.68 ± 0.18^b^
FFA (mmol/l)	1.26 ± 0.02^a^	1.63 ± 0.03^d^	1.57 ± 0.02^c^	1.50 ± 0.03^c^	1.52 ± 0.02^c^	1.32 ± 0.05^b^
Apo A-I (mg/dl)	35.24 ± 0.45^c^	29.02 ± 0.03^a^	32.84 ± 0.05^b^	33.51 ± 0.03^b^	33.69 ± 0.55^b^	35.87 ± 0.10^c^
Apo B (mg/dl)	7.84 ± 0.14^a^	14.35 ± 0.32^d^	9.99 ± 0.07^c^	8.59 ± 0.07^b^	8.78 ± 0.20^b^	8.10 ± 0.38^b^
Apo A-I/Apo B	5.14 ± 0.02^e^	2.64 ± 0.03^a^	3.15 ± 0.01^b^	4.21 ± 0.02^c^	4.26 ± 0.04^c^	4.53 ± 0.03^d^
ALT (Karmen/ml)	19.75 ± 0.21^a^	38.94 ± 0.09^c^	24.14 ± 0.05^b^	23.06 ± 0.06^b^	23.29 ± 0.18^b^	21.62 ± 0.05^b^
AST (Karmen/ml)	16.46 ± 1.10^a^	26.52 ± 0.17^b^	19.56 ± 0.10^a^	18.24 ± 0.25^a^	18.90 ± 0.50^a^	17.75 ± 0.90^a^

Mean ± SEM (*n* = 10). Means in the same row with different letters are significantly different at *p* < 0.05 by Tukey's range test. HTR (%) = (HDL − TC/TC) × 100; AI = [TC–(HDL − TC)]/HDL − C.

**Table 4:** Glucose, insulin, and adipokine levels in high-fat diet-fed mice orally administered with germinated and nongerminated Keunnunjami and brown rice extracts.

Parameter	Animal group					
	NC	HF	HF-B	HF-GB	HF-K	HF-GK
Initial glucose (mg/dl)	104.32 ± 1.06^a^	103.29 ± 1.01^a^	105.11 ± 1.11^a^	104.69 ± 1.30^a^	103.59 ± 1.17^a^	104.96 ± 1.66^a^
Final glucose (mg/dl)	105.69 ± 1.43^a^	145.96 ± 1.87^e^	137.99 ± 1.36^d^	132.65 ± 1.05^c^	132.19 ± 1.88^c^	125.36 ± 1.08^b^
Insulin (ng/ml)	1.24 ± 0.05^a^	2.24 ± 0.11^e^	1.88 ± 0.08^d^	1.63 ± 0.05^c^	1.57 ± 0.08^c^	1.41 ± 0.07^b^
Adiponectin (ng/ml)	0.82 ± 0.05^c^	0.31 ± 0.09^a^	0.56 ± 0.04^b^	0.62 ± 0.02^b^	0.81 ± 0.05^c^	0.91 ± 0.03^d^
Resistin (ng/ml)	24.00 ± 0.38^a^	32.23 ± 0.46^d^	28.80 ± 0.26^c^	26.76 ± 0.43^b^	25.45 ± 0.21^b^	23.09 ± 0.30^a^
TNF-*α* (*μ*g/ml)	5.63 ± 0.01^b^	10.12 ± 0.05^e^	7.99 ± 0.05^d^	6.85 ± 0.05^c^	6.92 ± 0.12^c^	5.49 ± 0.01^a^
HOMA-IR	1.65 ± 0.08^a^	8.74 ± 0.12^e^	5.18 ± 0.22^d^	3.72 ± 0.18^c^	3.67 ± 0.15^c^	2.68 ± 0.09^b^

Mean ± SEM (*n* = 10). Means in the same row with different letters are significantly different at *p* < 0.05 by Tukey's range test.

**Table 5:** Activities of lipid-regulating enzymes and *β*-oxidation in high-fat diet-fed mice orally administered with germinated and nongerminated Keunnunjami and brown rice extracts.

Parameter	Animal group					
	NC	HF	HF-B	HF-GB	HF-K	HF-GK
Hepatic enzyme activity (*μ*mol/min/mg protein)						
FAS	8.69 ± 0.10^a^	16.88 ± 0.18^d^	14.30 ± 0.20^c^	11.65 ± 0.32^b^	12.17 ± 0.48^b^	9.72 ± 0.43^a^
G6PD	4.39 ± 0.11^a^	8.27 ± 0.16^d^	6.82 ± 0.25^c^	6.24 ± 0.16^c^	6.22 ± 0.10^c^	5.32 ± 0.12^b^
CPT	29.61 ± 1.10^e^	11.02 ± 0.29^a^	14.38 ± 0.67^b^	17.90 ± 0.20^c^	15.72 ± 0.36^bc^	20.80 ± 0.53^d^
*β*-Oxidation	2.64 ± 0.05^e^	0.89 ± 0.03^a^	1.18 ± 0.02^b^	1.55 ± 0.03^c^	1.75 ± 0.05^d^	2.63 ± 0.50^e^
Adipocyte enzyme activity (*μ*mol/min/mg protein)						
FAS	16.51 ± 0.43^a^	37.43 ± 1.56^d^	30.30 ± 1.58^c^	28.27 ± 1.71^c^	21.27 ± 0.42^b^	18.06 ± 1.08^a^
G6PD	55.62 ± 1.45^a^	143.53 ± 1.44^e^	95.93 ± 1.53^d^	88.71 ± 0.95^c^	93.49 ± 1.43^d^	78.60 ± 0.43^b^
CPT	32.89 ± 0.63^e^	17.50 ± 0.70^a^	20.38 ± 0.48^b^	23.31 ± 0.22^c^	21.15 ± 0.90^bc^	26.74 ± 0.28^d^
*β*-Oxidation	3.13 ± 0.02^f^	1.52 ± 0.05^a^	1.87 ± 0.03^b^	2.35 ± 0.03^c^	2.57 ± 0.04^d^	2.90 ± 0.05^e^

Mean ± SEM (*n* = 10). Means in the same row with different letters are significantly different at *p* < 0.05 by Tukey's range test.

**Table 6:** Activities of glucose-regulating enzymes in high-fat fed mice orally administered with germinated and nongerminated Keunnunjami and brown rice extracts.

Parameter	Animal group					
	NC	HF	HF-B	HF-GB	HF-K	HF-GK
Hepatic enzyme activity (nmol/min/mg protein)						
GK	5.34 ± 0.09^e^	3.45 ± 0.02^a^	3.82 ± 0.02^b^	4.00 ± 0.03^b^	4.33 ± 0.02^c^	4.78 ± 0.06^d^
PEPCK	6.42 ± 0.02^a^	9.31 ± 0.09^e^	8.33 ± 0.08^d^	7.46 ± 0.10^c^	7.32 ± 0.08^c^	6.95 ± 0.05^b^
G6pase	62.52 ± 0.62^a^	82.30 ± 0.45^d^	77.09 ± 0.42^c^	71.02 ± 0.67^b^	68.91 ± 0.28^b^	63.10 ± 0.87^a^
Nephritic enzyme activity (nmol/min/mg protein)						
GK	12.35 ± 0.10^e^	8.35 ± 0.05^a^	9.62 ± 0.11^b^	9.87 ± 0.13^b^	10.39 ± 0.09^c^	11.57 ± 0.07^d^
PEPCK	13.68 ± 0.22^a^	36.03 ± 0.30^f^	30.04 ± 0.29^e^	25.82 ± 0.35^d^	21.06 ± 0.32^c^	17.99 ± 0.28^b^
G6pase	76.38 ± 0.50^a^	91.88 ± 0.43^e^	87.68 ± 0.34^d^	83.12 ± 0.67^c^	81.36 ± 0.21^c^	78.76 ± 0.34^b^

Mean ± SEM (*n* = 10). Means in the same row with different letters are significantly different at *p* < 0.05 by Tukey's range test.

## Data Availability

The data used to support the findings of this study are available from the corresponding author upon request.
